# Development and Field Validation of a Bivalve-Inspired Nonprehensile End-Effector for Citrus Harvesting

**DOI:** 10.3390/biomimetics11100758

**Published:** 2026-10-20

**Authors:** Yi-Han Chen, Jianhong Liang, Bingrun Chen, Jianan Zhai, Yuren Ban, Zijian Nie, Heng Li, Yitong Wu, Jixiang Zheng

**Affiliations:** 1 School of Electrical and Electronic Engineering, Nanyang Technological University, Singapore 639798, Singapore; 2 School of Mechanical Engineering and Automation, Beihang University, Beijing 100191, China; 3 Hangzhou International Innovation Institute, Beihang University, Hangzhou 311115, China; 4 Institute of Artificial Intelligence, Beihang University, Beijing 100191, China; 5 Guangxi Beitou IT Innovation Technology Investment Group Co., Ltd., Nanning 530200, China; 6 Guangxi Zhuang Autonomous Region Fruit Technical Guidance Station, Nanning 530200, China

**Keywords:** biomimetic end-effector, bivalve-inspired mechanism, citrus harvesting, pedicel cutting, teleoperation, fruit damage

## Abstract

Fruit-body grasping can expose citrus peel to concentrated loads during robotic harvesting. This study develops a bivalve-inspired end-effector that combines opposing C-ring closure, pedicel cutting, and passive fruit reception without deliberately clamping the fruit body. Paired shell closure informed the mechanism, while the ring dimensions were selected to accommodate the target fruit sizes and provide access to the pedicel region. An idealized normal-contact analysis provided supporting mechanical interpretation of the concave inner surface. The end-effector was integrated with a four-degree-of-freedom positioning platform and a vision-assisted, operator-supervised teleoperation system. The operator aligned the closure plane, commanded reduced-output engagement and primary cutting, and applied wrist rotation only when the fruit remained attached. In a commercial Wogan mandarin orchard, 85 engaged harvesting outcomes were recovered and inspected after the cutting and reception sequence. Seventy fruits were intact and three had superficial grazes, giving an acceptable-outcome proportion of 85.9% under the study’s visual inspection criteria. The remaining outcomes comprised six ring-tip punctures and six fruit-body mis-cuts. A subset of 14 harvested fruits had a median residual pedicel length of 11.0 mm and a range of 6.0–40.8 mm. These results establish the feasibility of the integrated mechanism under teleoperation and identify closure-plane placement, ring-tip contact, and cutting consistency as priorities for further development.

## 1. Introduction

Robotic fruit harvesting requires reliable target localization, access through the canopy, controlled detachment, and careful handling of the harvested fruit. Systems developed for citrus, apples, strawberries, and kiwifruit illustrate the continuing difficulty of integrating these tasks in orchards and protected cultivation [1,2,3,4]. For citrus, maintaining peel integrity is particularly important: mechanical injury can rupture oil cells, damage surrounding tissue, and increase susceptibility to decay [5,6]. The end-effector must therefore separate the pedicel while limiting harmful contact with the fruit.

Grasping-based approaches use the fruit body to transmit detachment forces. Compliant fingers, soft materials, and sensing can improve this interaction. Examples include sensorized raspberry harvesting [7], soft apple grippers [8], soft grippers for smaller fruit [9], and flexible citrus grippers [10]. Vacuum end-effectors offer another way to hold fruit during removal [11]. These developments address different crops and operating conditions, so their reported success rates cannot be compared without considering target selection, task definitions, and damage criteria.

Directing separation forces towards the stalk is also an established design direction. Feng et al. [12] combined stalk holding and cutting for cherry-tomato bunches, while Quaglia et al. [13] developed an interchangeable underactuated tool that holds and cuts grape peduncles. Parsa et al. [14] combined stem gripping and cutting with separator fingers that clear nearby obstructions during strawberry picking, avoiding direct contact with the target fruit flesh. Park et al. [15] integrated cutting, suction, and transport modules in a tomato-harvesting end-effector. Zhao et al. [16] investigated deployable scissor mechanisms for enclosing and retaining fruit. Kondo et al. [17] used main-stem guidance and peduncle sensing to harvest tomato clusters without prior knowledge of peduncle orientation. In citrus, Wang et al. [18] developed a bite-mode end-effector and optimized its posture for variable stalk orientations. Together, these studies show that stalk cutting, reduced fruit-body grasping, and accommodation of variable stalk orientation provide a substantial foundation for further mechanism development.

The present design addresses the harvesting of individual citrus fruits through two opposing C-shaped rings. The rings approach around the upper fruit profile and intercept the pedicel within their closure region. Their cutting edges apply the primary separation load to the pedicel, and a net receives the detached fruit. This arrangement reduces the need to orient a narrow cutter along an explicitly estimated pedicel axis. It still requires the pedicel to cross the closure region and the closure plane to remain clear of the fruit body.

The mechanical reference is the paired closure of bivalve shells: two curved rigid members move about a shared hinge under actuator loading. Here, that arrangement is adapted for pedicel engagement and cutting. If the primary cut is incomplete, wrist rotation supplements ring closure to complete separation. The contribution lies in combining this C-ring mechanism with staged actuation, conditional wrist rotation, and passive reception for citrus harvesting. The principal evidence is the mechanical design and its field evaluation; contact analysis and the vision-assisted positioning platform support that evaluation.

Accordingly, this paper describes the biological reference and engineering design, examines idealized normal contact with the concave ring surface, and evaluates fruit damage and residual pedicel length during teleoperated orchard harvesting. The comparison with related mechanisms in Section 6 places the design in the context of existing non-grasping and reduced-grasping approaches.

## 2. Biomimetic Design and Morphological Mapping

### 2.1. Biological Reference and Shell Measurements

**Figure 1:**
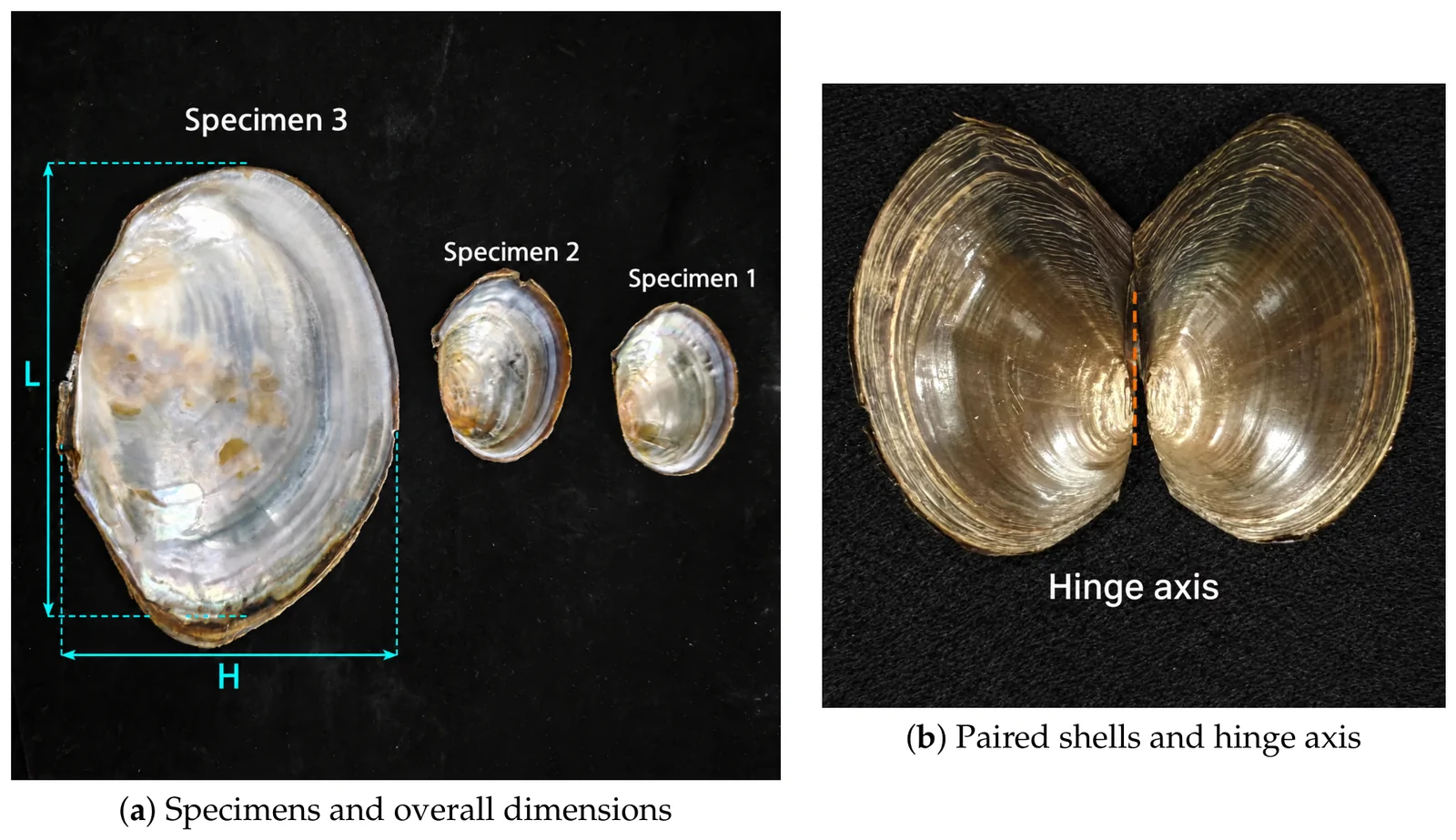
Shell specimens used as the biological reference. (**a**) Specimens 1, 2, and 3 denote the smallest, intermediate, and largest specimens, respectively. In the displayed orientation, *L* is the maximum top-to-bottom length and *H* the maximum left-to-right width; dashed extension lines identify the corresponding extremal points. (**b**) The paired shells show the hinge axis.

Three freshwater mussel specimens identified as *Sinanodonta woodiana*, a member of the Chinese pond-mussel species complex [19], were obtained from a local aquatic market. Their rounded outlines, concave inner surfaces, and paired-hinge arrangement provided an accessible morphological reference for a mechanism intended to surround rounded citrus fruits. The biological reference was limited to this structural arrangement and closure motion; the end-effector was not intended to reproduce the shell material or the complete biological function. The shells were opened to expose their inner surfaces and hinge regions (Figure 1).

Bivalve adductor muscles connect to the inner shell surfaces and generate valve closure [20]. The relevant engineering features are the two rigid members, shared hinge, opposing motion, and concave inner geometry. Shell morphology can affect mechanical response [21], but the engineering device employs different materials and serves a different function: its meeting edges cut the pedicel.

The measured overall dimensions and inner curvature estimates are listed in Table 1. Shell length *L* and width *H* were the maximum dimensions defined in Figure 1a. For a selected equatorial inner-profile section, the chord length *C* was the straight-line distance between the section endpoints, and the sagitta *h* was the greatest perpendicular distance from that chord to the inner surface. Approximating this section by a circular arc gives

(1) $$R_{\mathrm{inner}}=\frac{C^{2}}{8h}+\frac{h}{2}.$$

The sagitta describes the depth of the inner profile; it is distinct from the overall width *H* visible in the photograph.

The normalized radii describe the curvature proportions of these three specimens. They support the use of a concave inner profile as a design reference, but do not prescribe an absolute ring size. The C-ring radius was selected from the target fruit dimensions and access requirements, as described below.

### 2.2. Morphological Mapping and Design Requirements

**Figure 2:**
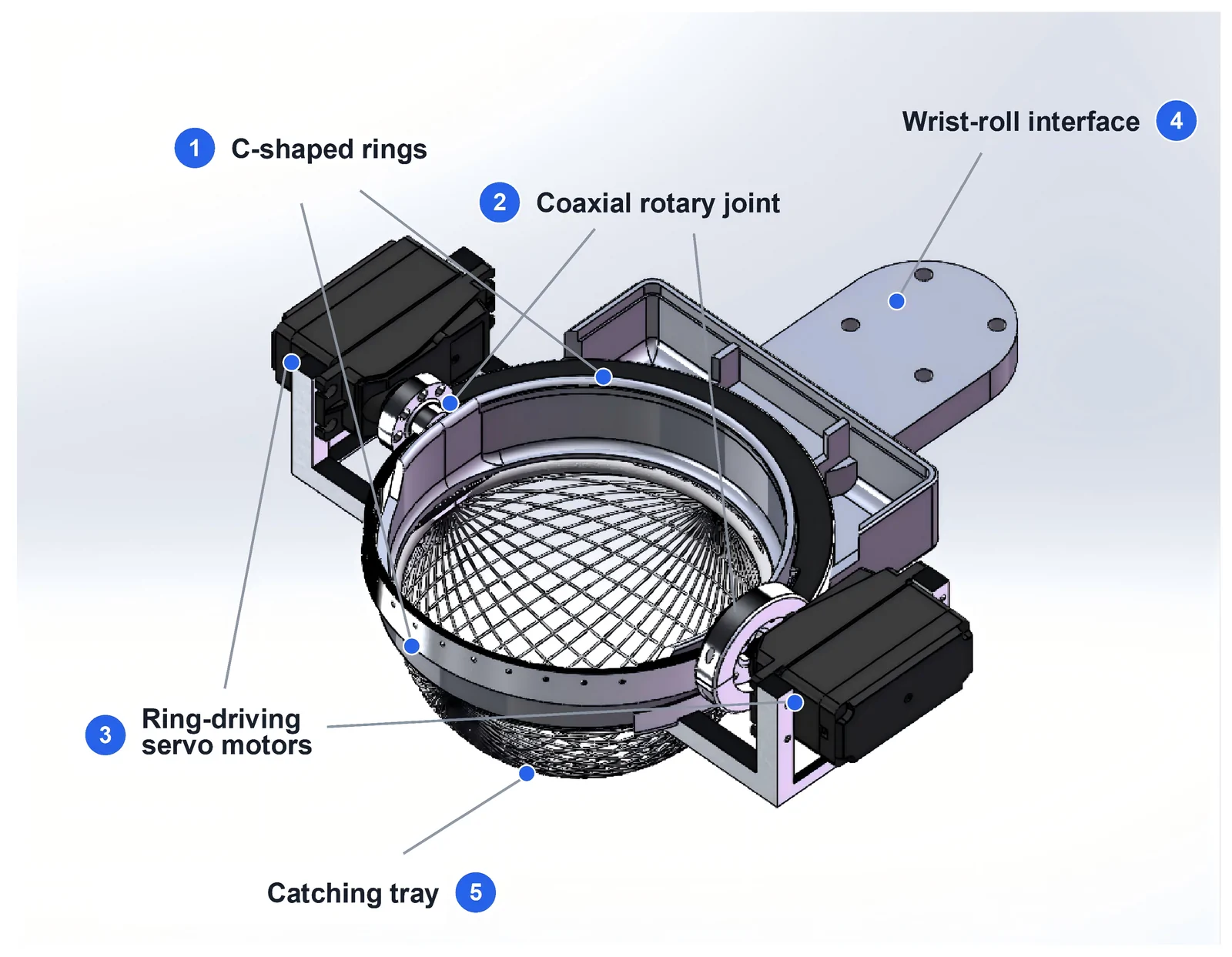
Numbered components of the C-ring end-effector, corresponding to Table 2: (1) C-shaped rings; (2) coaxial rotary joint; (3) ring-driving servo motors; (4) wrist-roll interface; and (5) elastic fruit-catching net, labelled *catching tray* in the CAD view.

The design requirements were to admit the upper part of an individual citrus fruit, intercept its pedicel without deliberately squeezing the fruit body, apply a cutting action, and retain the fruit after separation. Table 2 connects these requirements to the selected biological features and the numbered engineering components in Figure 2.

The mapping transfers a structural arrangement and motion principle. Staged actuator commands, cutting edges, wrist rotation, and fruit reception are engineering adaptations. The measured shell proportions provide context for the concave profile, while the harvesting task determines the working dimensions.

### 2.3. End-Effector Geometry and Motion

The end-effector comprises two semicircular alloy rings (component (1)), a coaxial rotary joint (2), and two ring-driving servo motors (3), mounted on a frame connected to the manipulator wrist (4). Each ring has an inner radius of 40.1 mm, a wall thickness of 4 mm, and an axial width of 20 mm. Each servo has a stated torque rating of 150 kgf·cm, equivalent to approximately 14.7 N·m. Polymer guards cover the ring-tip regions to soften incidental contact, while the opposing cutting edges remain available for pedicel severance.

The 40.1 mm inner radius was selected for a target equatorial fruit diameter of approximately 60–80 mm. Smaller rings would exclude larger fruits; substantially larger rings would increase the required canopy clearance and could place the cutting region farther from the calyx, leaving a longer residual pedicel. Most fruits encountered during operation were away from the upper size limit. A fruit radius of 40 mm is therefore an upper design boundary rather than a representative radius for the orchard.

For fruit radii of 30–40 mm, the ratio of ring radius to fruit radius ranges from approximately 1.34 to 1.00. This is a geometric accommodation range, not a direct scale copy of the shell measurements. At the largest equatorial section, the nominal radial clearance is only 0.1 mm. During approach, the rings are placed around the narrower upper fruit profile near the pedicel, so the usable clearance depends on fruit shape, orientation, and closure-plane height. The intended cutting location is approximately 10 mm above the calyx. This is a device positioning target; subsequent trimming may still be needed for handling.

**Figure 3:**
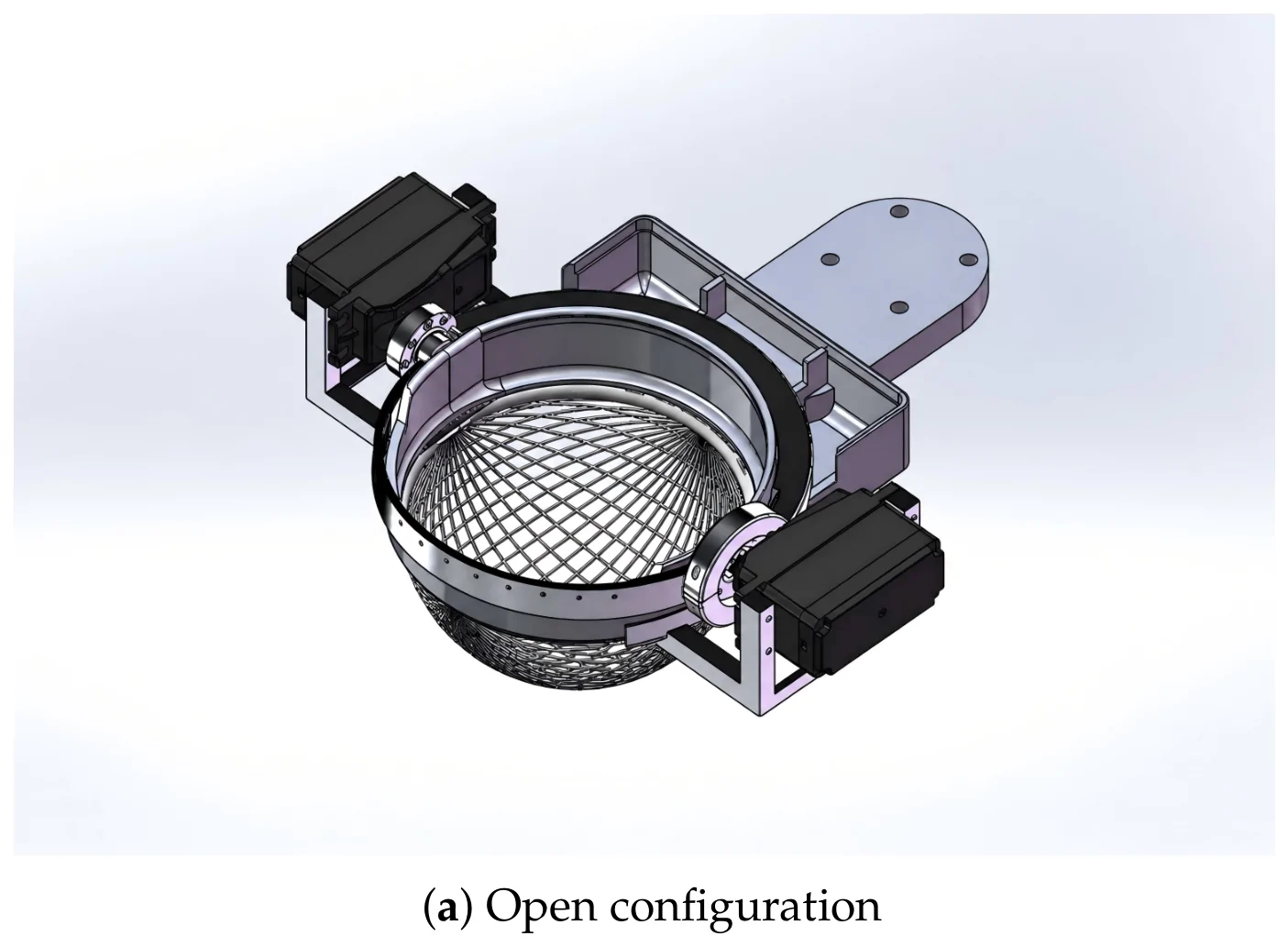

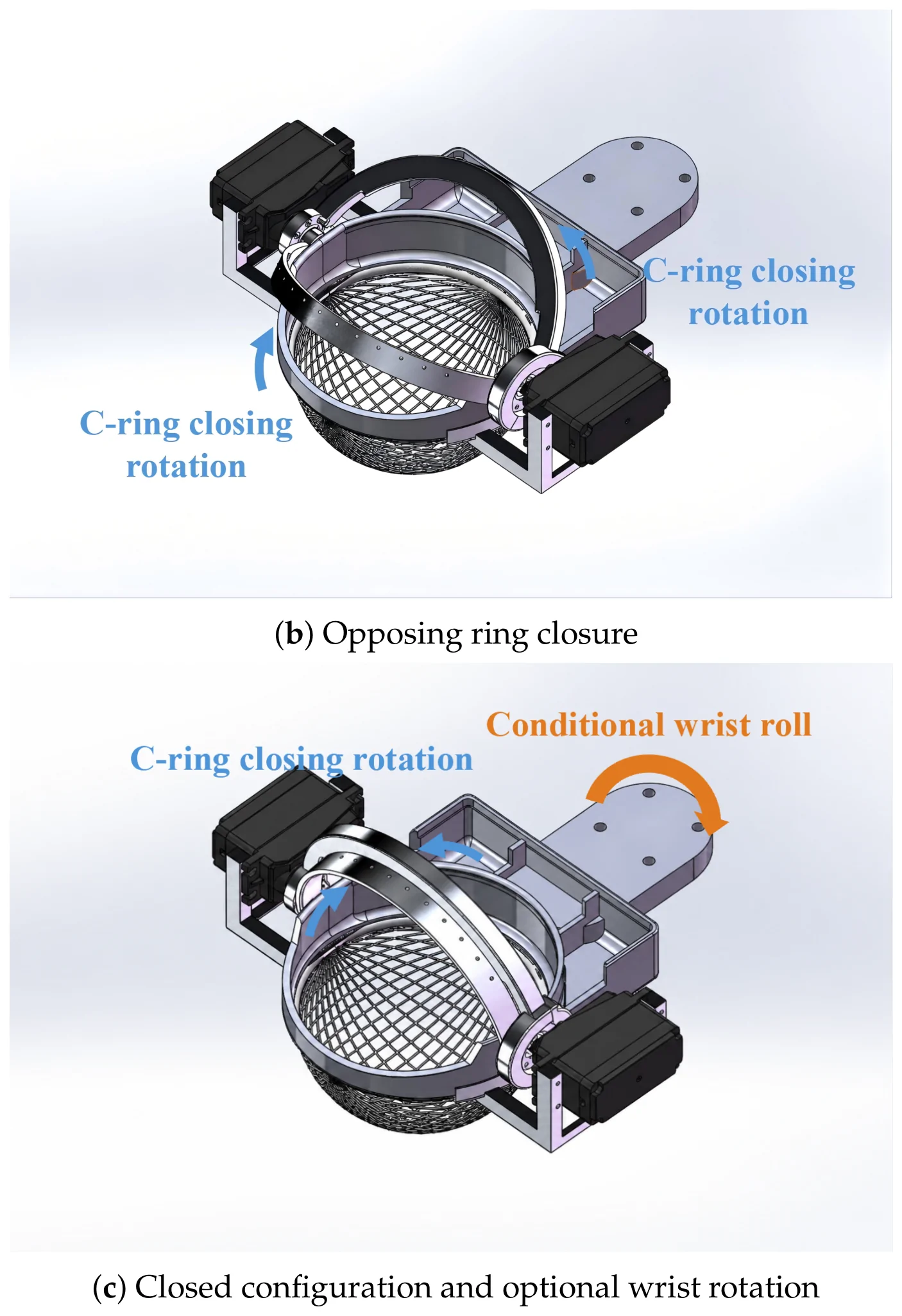
CAD views of the mechanism during closure. Blue arrows indicate C-ring closing rotation about the coaxial joint. The orange arrow indicates wrist rotation of the assembly, used only when the primary cut leaves the fruit attached.

Figure 3 shows the open, intermediate, and closed configurations. Each servo rotates its ring about the shared axis, bringing the opposed edges into the pedicel region. A pedicel crossing this region can be intercepted during closure without an explicit estimate of its three-dimensional axis. Placing the closure plane below the pedicel, however, can bring the edges into the fruit body. The staged operating sequence is described in Section 4, with representative orchard sequences in Figure 6.

A spherical elastic catching net (component 5) is mounted below the rings and receives the fruit after separation. The deepest point of the unloaded net is approximately 60 mm below the cutting region. Because this distance is comparable to the vertical dimension of the target fruit, the fruit is already within the compliant receiving region before complete detachment. After pedicel separation, the net supports and retains the fruit. The net serves the reception function; the C-ring edges provide the intended pedicel separation action.

## 3. Idealized Normal-Contact Analysis of the Concave C-Ring

A concave inner surface is useful when ring closure produces incidental contact with the rounded peel. Published citrus clamping studies demonstrate that material properties and contact geometry affect tissue stresses [22]. Here, the analysis examines the normal-contact response of the ring geometry as a supporting design consideration.

In a section through the ring curvature, a spherical fruit of radius $R_{c}$ contacting a concave surface of radius $R_{b}>R_{c}$ has relative curvature

(2) $$\kappa_{\mathrm{rel}}=\frac{1}{R_{c}}-\frac{1}{R_{b}}.$$

A smaller curvature mismatch permits a more conforming contact along that section [23]. The actual ring has finite width and a second principal curvature, so this sectional relation does not determine a three-dimensional pressure-reduction factor.

A static finite-element model in SolidWorks Simulation 2024 represented the C-rings with their specified dimensions and the fruit as a homogeneous oblate ellipsoid with equatorial and polar semi-axes of 38 and 33 mm, respectively. The fruit elastic modulus was 0.6 MPa, rounded from the approximately 0.584 MPa whole-orange value reported by Namdari Gharaghani and Maghsoudi [24]. A Poisson’s ratio of 0.45 was an effective-material modelling assumption. The ring components were assigned AISI 1020 steel properties (E=200 GPa, ν=0.29) to approximate a stiff surface relative to the fruit. This idealization represents metal–fruit contact without a separate compliant guard layer.

Frictionless surface-to-surface contact isolated the normal component. A small basal fruit region was constrained to suppress rigid-body motion, with soft-spring stabilization during contact establishment. Nominal total normal loads of 10 and 20 N were prescribed along the closing direction. The 10 N case served as the baseline and the 20 N case as a twofold-load sensitivity case under otherwise identical conditions, allowing the change in the predicted contact region and stress field to be examined. The prescribed loads were analysis inputs rather than measured harvesting forces. Curvature-based meshing used a nominal element size of 1–3 mm, with local contact-region refinement to approximately 0.3 mm.

**Figure 4:**
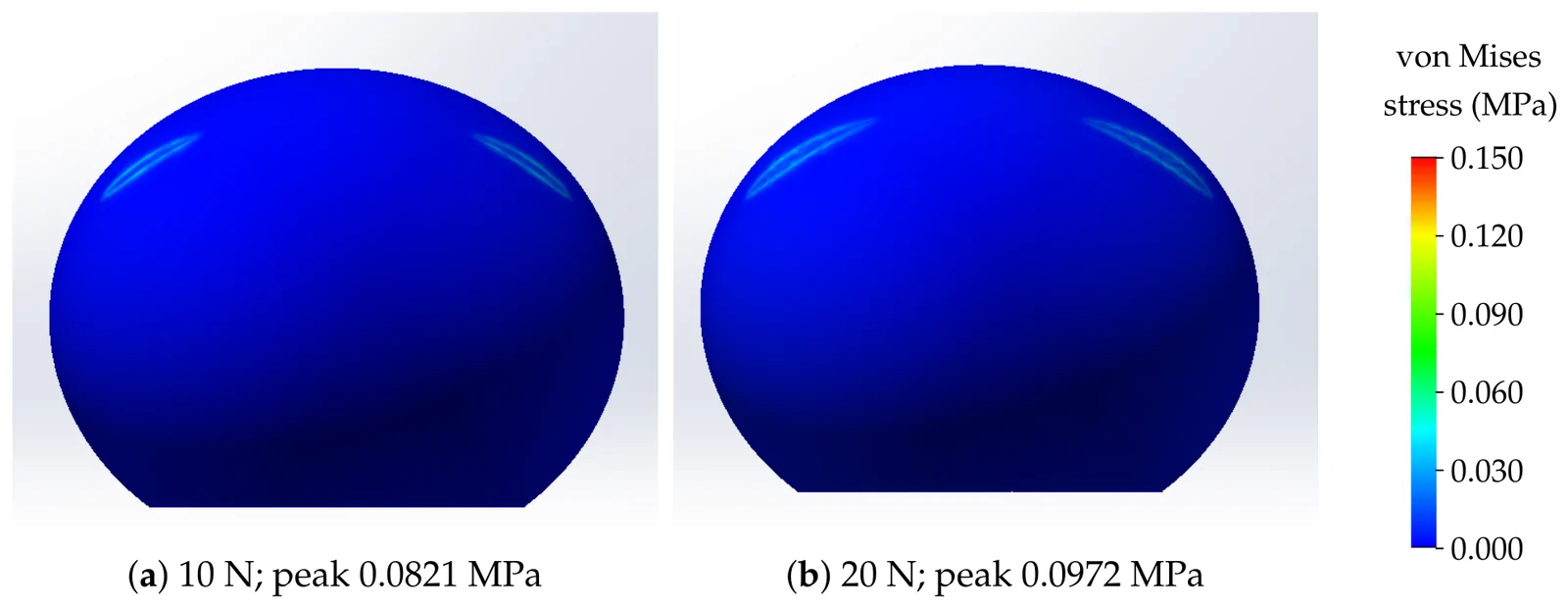
Finite-element results for idealized C-ring normal contact. Both cases use the same 0–0.15 MPa colour scale. The original contours and colour strip are retained; the stress labels have been enlarged and converted from Pa to MPa.

The predicted peak nodal von Mises stresses were 0.0821 and 0.0972 MPa at 10 and 20 N, respectively (Figure 4). The contours show contact extending along the curved ring region. These results describe the selected geometry, effective material, and boundary conditions. Von Mises stress is an equivalent internal stress measure, not contact pressure or a validated peel-damage threshold.

The analysis supports a mechanical interpretation of the concave surface under incidental normal contact. Closure-plane displacement towards the fruit body changes the interaction to edge cutting; ring-tip penetration and tangential grazing likewise involve different contact and failure processes. Those modes are assessed from the field observations below. A model of normal contact alone cannot predict their occurrence.

## 4. Harvesting Strategy and Teleoperated Testbed

### 4.1. Three-Stage Harvesting Strategy

**Figure 5:**
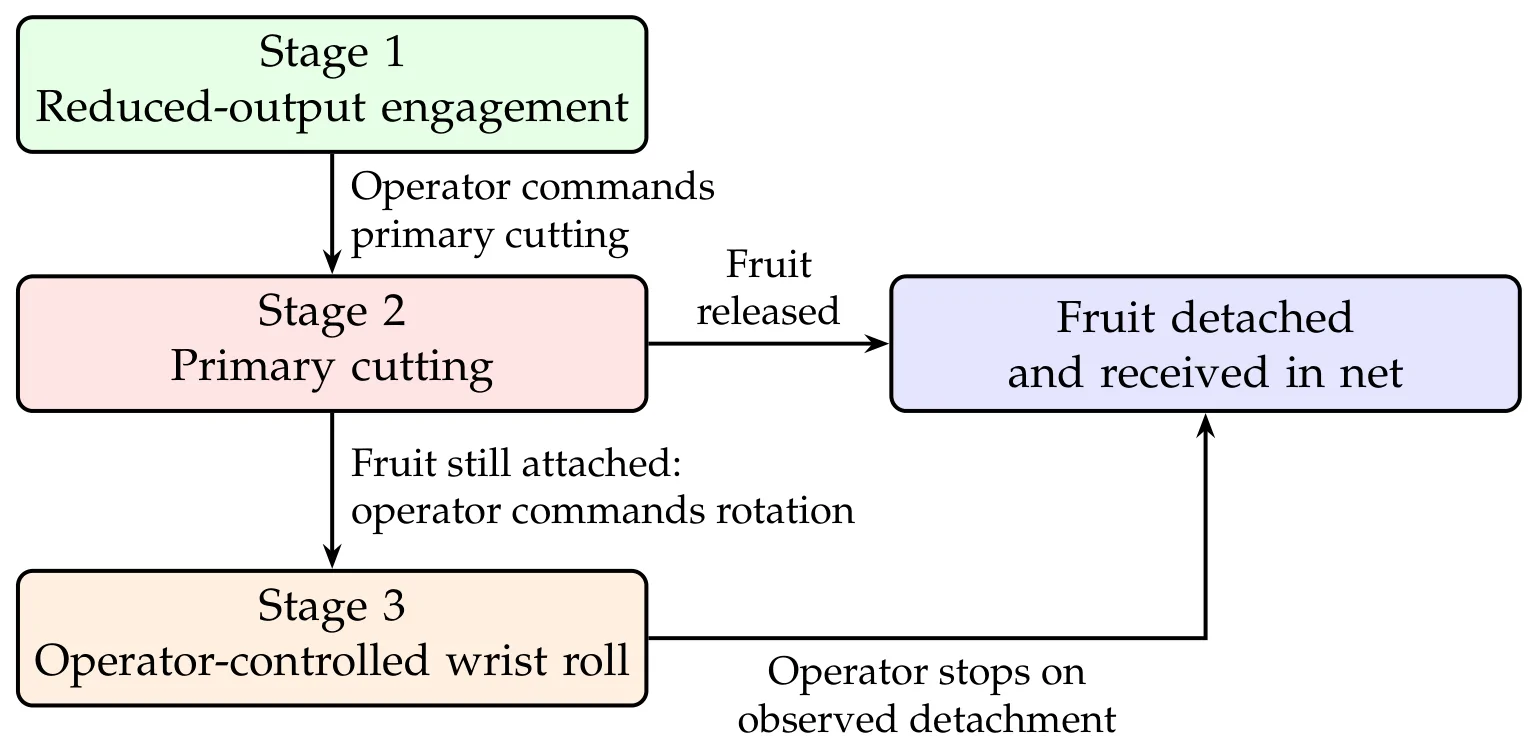
Harvesting sequence under operator supervision. Stage transitions follow manual commands and visual assessment of engagement and detachment. Wrist rotation is omitted when primary cutting releases the fruit.

The mechanism uses reduced-output engagement, higher-output primary cutting, and conditional wrist rotation. The operator initiates each stage and judges whether separation has occurred (Figure 5).

*Stage 1: reduced-output engagement.* The operator uses the live camera view and initial fruit-position reference to place the closure plane near the pedicel, then commands the rings to close at the reduced actuator setting. The initial reference is obtained from a CBAM-YOLOv8 fruit bounding box and the corresponding Gemini 336L depth measurement, as detailed in Section 4.3. After visually confirming the engagement configuration, the operator manually commands the primary cut. A fixed elapsed time does not trigger the transition.

*Stage 2: primary cutting.* The operator switches the ring-driving servos to the preset higher-output setting, and the opposing edges continue across the pedicel. If the cut releases the fruit, it is received and retained by the elastic net. The manipulator does not intentionally pull the fruit down during this stage. The two output levels are preset servo commands, and the operator switches between them manually after visually confirming engagement; elapsed time, force sensing, and torque feedback do not trigger the transition.

*Stage 3: conditional wrist rotation.* If the fruit remains attached after primary cutting, the operator maintains C-ring closure and continuously controls wrist rotation until detachment is observed. The fruit’s loss of connection to the tree and its reception by the net provide the stopping cues. The approximate wrist rotation typically used in these operations was 60–90°. This range describes operator-controlled motion ending at separation, rather than a predetermined target angle for each fruit. Wrist rotation applies torsion and bending through the engaged pedicel region to assist completion of an incomplete cut.

**Figure 6:**
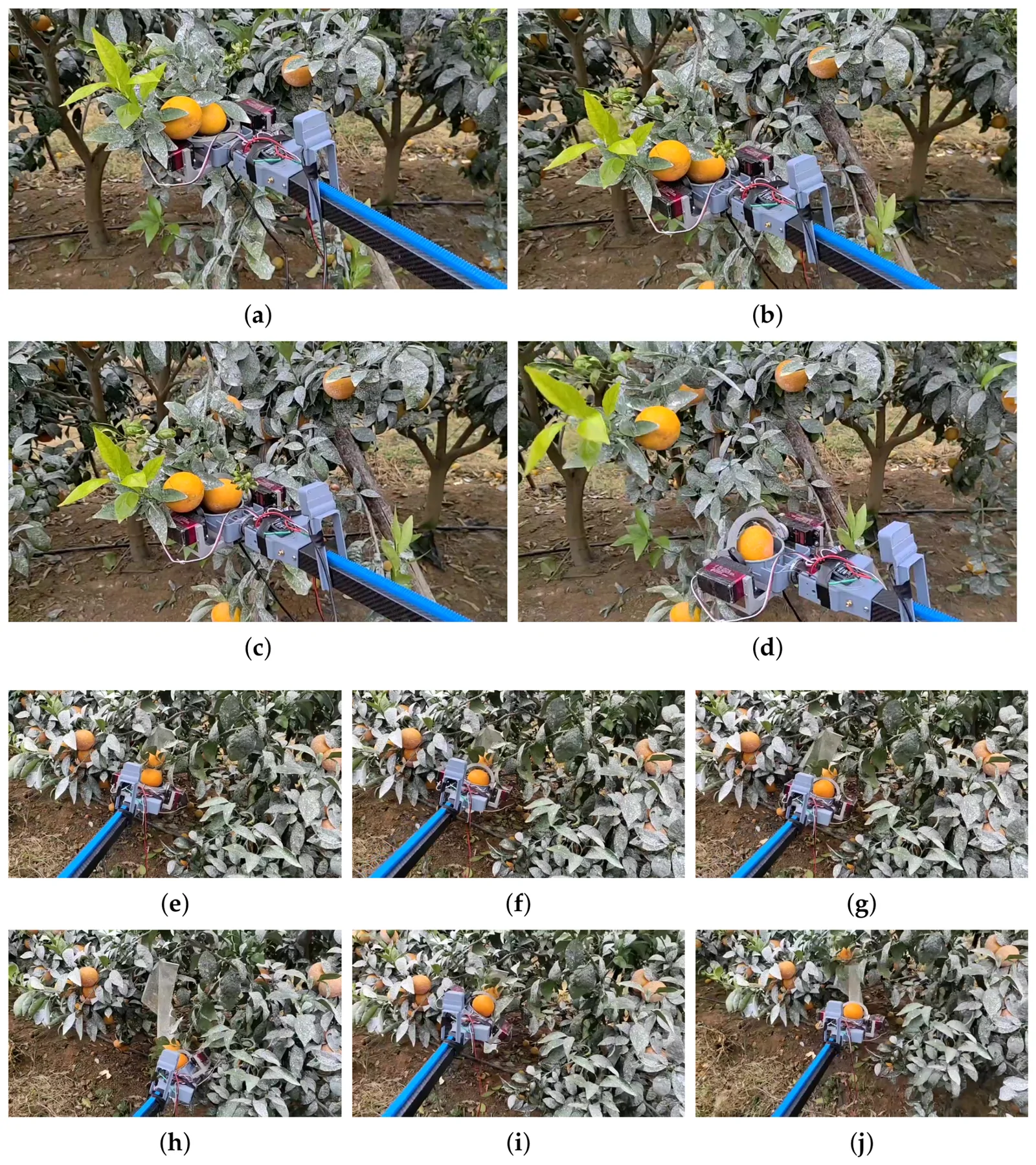
Representative orchard harvesting sequences. (**a**–**d**) Direct separation: (**a**) approach; (**b**) partial C-ring engagement; (**c**) full C-ring closure and primary cutting; and (**d**) fruit reception in the elastic net. (**e**–**j**) Wrist-assisted separation: (**e**) engagement; (**f**) primary cutting; (**g**) maintained C-ring closure; (**h**) operator-controlled wrist rotation; (**i**) fruit separation; and (**j**) fruit reception in the elastic net. Each sequence is arranged chronologically from left to right and top to bottom.

Figure 6 illustrates the direct and wrist-assisted detachment paths observed in the orchard.

### 4.2. Four-DOF Positioning Platform

**Figure 7:**
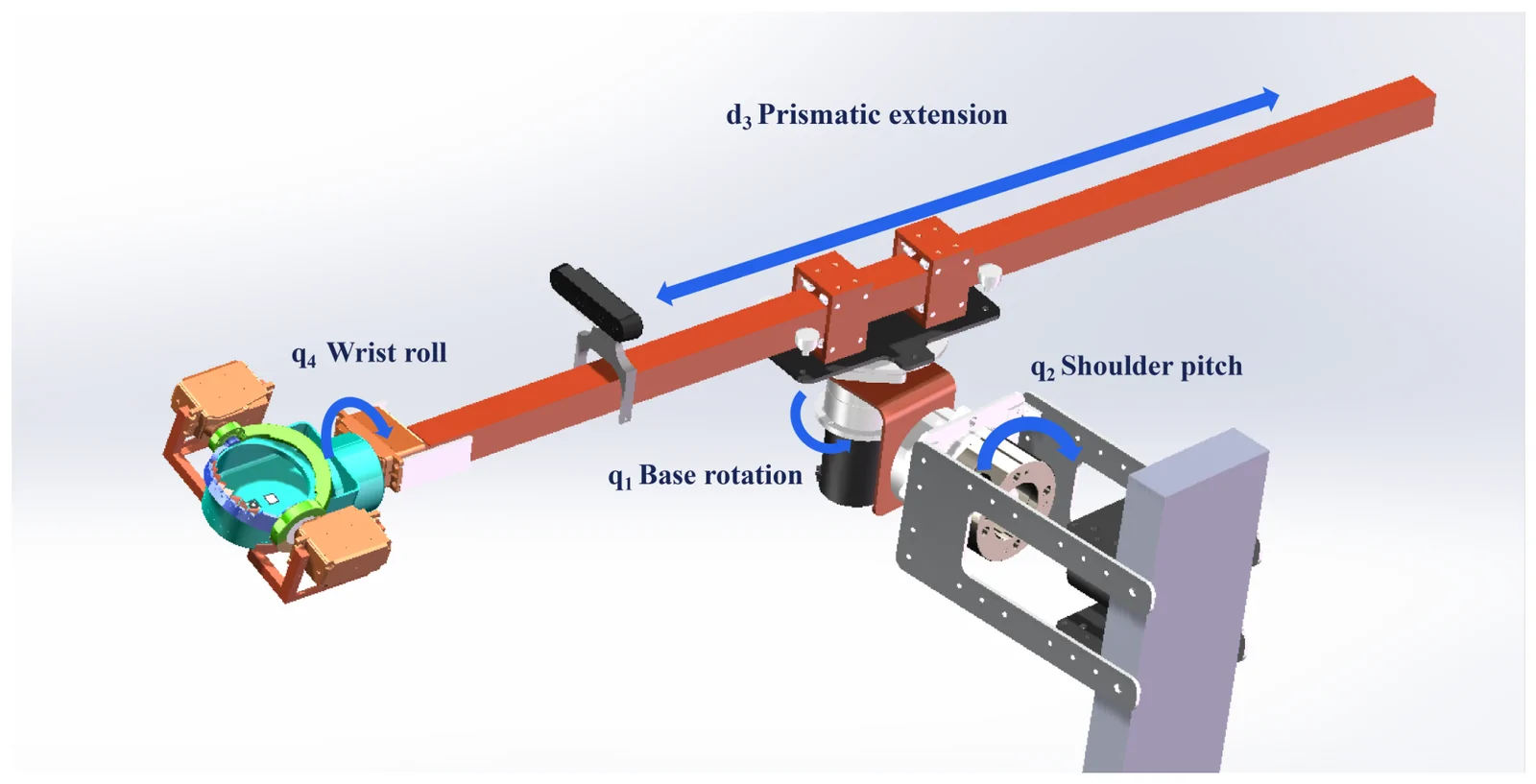
Four-DOF positioning platform. Labels and arrows indicate base rotation $q_{1}$, shoulder pitch $q_{2}$, radial extension $d_{3}$, and wrist roll $q_{4}$. The distal C-ring closure mechanism is shown separately in Figure 2 and Figure 3.

The end-effector is mounted on a four-degree-of-freedom manipulator carried by an operator-controlled mobile platform. The manipulator comprises base rotation $q_{1}$, shoulder pitch $q_{2}$, radial extension $d_{3}$, and wrist roll $q_{4}$ (Figure 7). The first three variables position the end-effector; $q_{4}$ changes its roll orientation and supplies the optional auxiliary separation motion. The two C-ring drives belong to the end-effector and are additional to these four manipulator degrees of freedom.

The positioning ranges used in the kinematic model were $-40^{\circ }\leq q_{1}\leq 40^{\circ }$, $45^{\circ }\leq q_{2}\leq 135^{\circ }$, and $0\leq d_{3}\leq 0.8$ m. The $q_{1}$ and $q_{2}$ intervals are commanded working ranges used for workspace calculation rather than mechanical stops, whereas 0.8 m is the available radial-extension stroke. With the fixed 0.5 and 0.2 m offsets in the Denavit–Hartenberg model, the maximum modelled distance from the base-frame origin to the wrist reference point is approximately 1.22 m. The first three variables therefore define an idealized spherical-sector positioning workspace. The motor-driven wrist joint $q_{4}$ has no fixed mechanical angle stop; its practical rotation is constrained by cable routing and possible structural interference. Wrist roll changes orientation without extending the positioning workspace, and the 60–90° motion reported above is the range typically used for auxiliary separation.

Standard Denavit–Hartenberg kinematics were used to compute commanded joint positions analytically. The inverse-kinematics solver and controller executed the operator’s motion commands. Joint velocity and acceleration scaling factors were set to 0.5 relative to the configured motion limits during approach and wrist rotation.

### 4.3. Vision-Assisted Target Detection

An Orbbec Gemini 336L active-stereo depth camera supplies colour and depth images. The manufacturer’s specifications give a depth field of view of approximately $90^{\circ }\times 65^{\circ }$, depth resolution up to 1280×800 at 30 frames/s, and an ideal operating range of 0.25–6 m [25]. The camera provides an initial position reference for approach.

The detector was developed using 9274 orchard images acquired in Guangxi, including changes in illumination, fruit scale, orientation, partial occlusion, and overlap. Fruit bounding boxes were annotated in Label Studio, images were preprocessed using OpenCV and resized to 640×640 pixels, and the dataset was divided into training, validation, and test subsets in a 7:2:1 ratio. A YOLOv8 detector incorporating the convolutional block attention module (CBAM) [26,27] was trained for 100 epochs with a batch size of 16. Development used Python 3.9.21 and PyTorch 2.6.0 under Ubuntu 20.04, with an AMD Ryzen 5800H processor and an NVIDIA GeForce RTX 3060 GPU for training. Camera acquisition used the Orbbec SDK. Motion commands were generated in a ROS-based environment and transmitted through the RK3588 controller and CANopen communication.

For a detected fruit, the bounding-box centre and its aligned depth value were back-projected using the camera intrinsics and transformed into the manipulator frame using the hand–eye calibration. The resulting coordinate supplied the initial approach reference. The detector identified fruit rather than pedicels; the operator adjusted the final closure plane using the live image.

### 4.4. Teleoperation Protocol

The operator controlled the mobile base, selected the target, commanded the initial approach, and adjusted the final end-effector pose. The controller handled coordinate transformation, inverse kinematics, and execution of the commanded motion. The operator then initiated engagement and cutting and decided whether wrist rotation was needed. There was no contact sensor in the catching net; engagement and separation were judged visually.

Before approach, the operator assessed whether the detected fruit had a sufficiently clear access corridor. Outer-canopy targets and available inter-branch gaps were preferred. When branches blocked the path or prevented placement of the pedicel within the closing region, the operator skipped the target or repositioned the platform. Physical access was therefore assessed separately from fruit detection. Automatic branch segmentation and collision-aware canopy planning were not implemented.

## 5. Experimental Validation

### 5.1. Orchard-Image Detection

The CBAM-augmented YOLOv8 detector achieved an mAP@0.5 of 82.3% on the held-out image test set. This is an aggregate two-dimensional object-detection metric at an intersection-over-union threshold of 0.5. Performance was not stratified by occlusion level, and three-dimensional localization error was not separately quantified.

**Figure 8:**
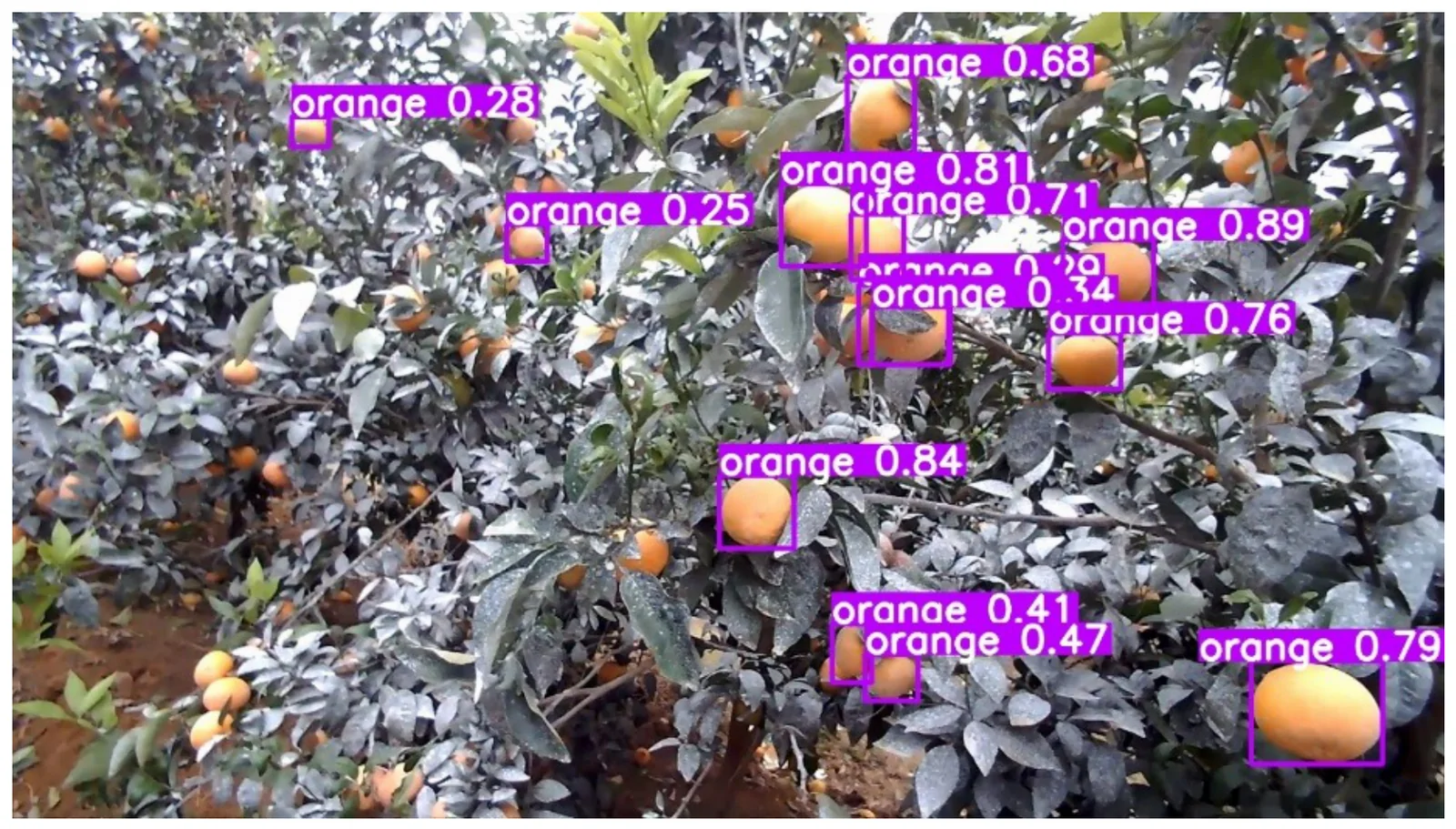
Representative orchard detection output used as the operator’s visual reference, showing fruit bounding boxes and confidence scores under variation in scale and partial occlusion.

Partially visible fruits could be detected from their remaining image features, as illustrated in Figure 8. Severe occlusion could still lead to missed detections or an unsuitable depth estimate. In field use, the operator inspected the detection overlay, selected an accessible target, and refined the approach pose from the live view.

### 5.2. Orchard Trials and Assessment Protocol

Field trials were conducted in a commercial Wogan mandarin orchard in Wuming District, Nanning, Guangxi (approximately 23.2° N), on slopes of 15–20°. Canopies extended approximately 0.8–2.5 m above ground. The operator moved the platform along the rows and guided radial extension through available canopy gaps. For reporting by fruit size, small fruits had equatorial diameters 60≤D<70 mm, and large fruits had diameters 70≤D≤80 mm.

An *engaged outcome* was an attempted harvest in which the C-rings reached the pedicel region and the primary cutting command was executed. The assessment included 85 recovered engaged outcomes. Targets rejected before engagement because of obstructed access were excluded, and the number of such rejected targets was not separately recorded. Thus, the assessed population represents the cutting and reception sequence after engagement.

All 85 outcomes were inspected after completion of the harvesting sequence. Detached fruits were examined after reception in the net and removal; separated fruit sections from mis-cuts were also recovered for assessment. Macroscopic inspection was followed by stereomicroscopy when surface abnormalities were present. An outcome was classified as acceptable if the fruit was intact or had only a superficial graze, without visible rind penetration, oil exudation, or fruit-body transection. Damage categories were assigned using both morphology and the corresponding field observations.

### 5.3. Fruit Damage

Three types of damage were observed. *Type A: fruit-body mis-cut* occurred when the closure plane intersected the fruit instead of the pedicel and the cutting edges passed through the rind and flesh. Six such outcomes were recorded, including two large and four small fruits. Field observations associated these events with incorrect closure-plane placement or fruit displacement during cutting, sometimes following contact with adjacent branches.

*Type B: ring-tip puncture* consisted of localized circular or oval rind penetrations, typically 1–3 mm across, consistent with ring-tip contact during approach or engagement. Six cases were recorded: five large fruits and one small fruit. Stereomicroscopy showed localized oil release at the breach sites.

*Type C: superficial graze* consisted of shallow linear marks approximately 5–15 mm long, consistent with tangential contact between a ring edge and the rind. Three cases were recorded: two large fruits and one small fruit. At 20× magnification, these marks showed no open rind breach or visible oil exudation and met the study’s acceptable-outcome criterion.

Of the 85 assessed outcomes, 70 fruits (82.4%) showed no visible fruit-body damage. Including the three superficial grazes, 73 outcomes (85.9%) met the specified acceptability criterion. The six mis-cuts and six punctures accounted for the remaining outcomes (Table 3). No characteristic damage attributable to net impact was observed, consistent with the close placement and compliance of the receiving net.

**Figure 9:**
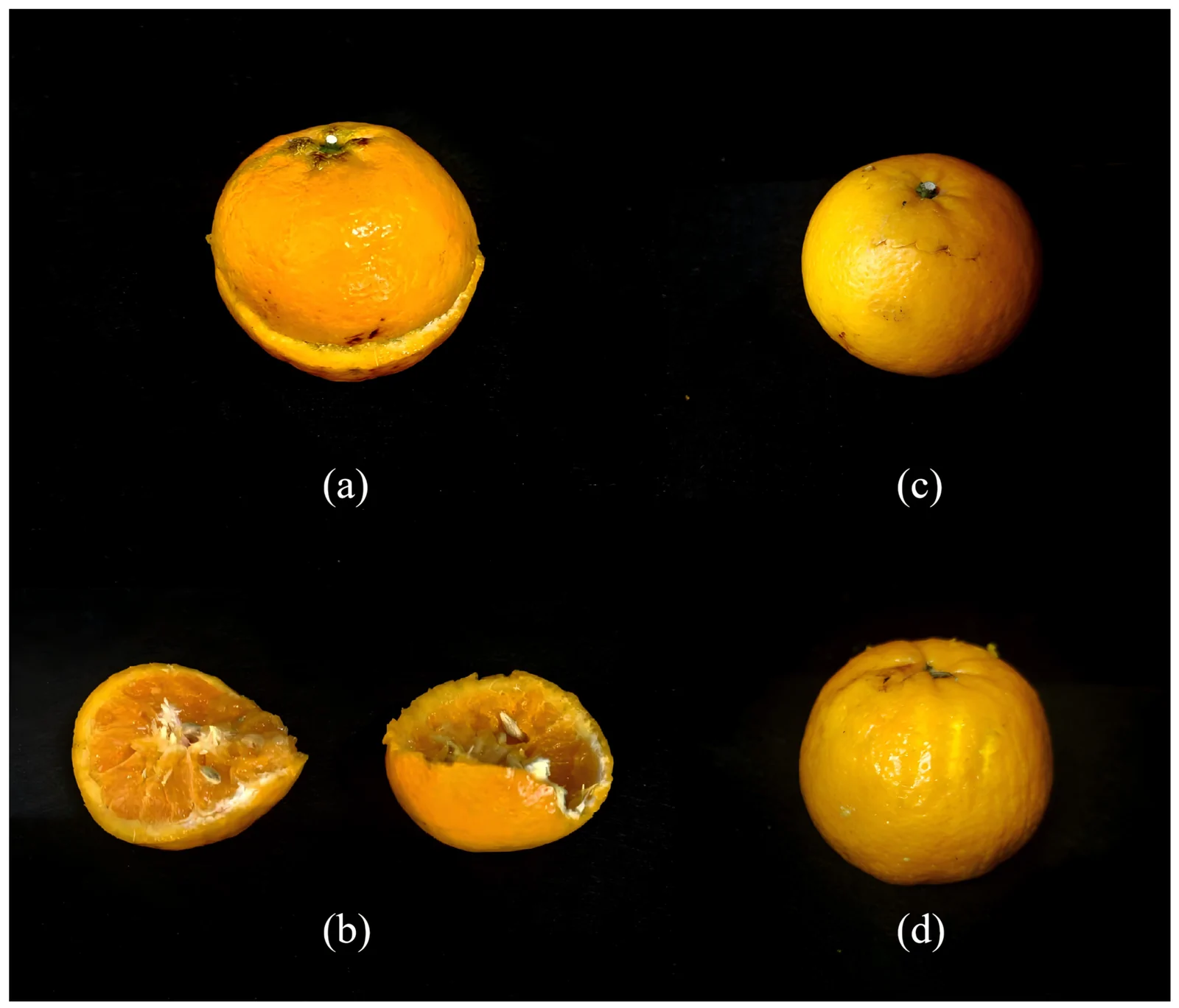
Representative field damage: (**a**) Type A mis-cut, external view; (**b**) Type A mis-cut, cross-section; (**c**) Type B ring-tip puncture with oil release; and (**d**) Type C superficial graze.

Figure 9 shows representative damage morphologies. The mis-cut views show complete fruit-body transection, whereas the puncture and graze images show localized contact damage.

### 5.4. Residual Pedicel Length

Residual pedicel length was measured on a randomly selected subset of 14 harvested fruits. The mean was 13.7 mm, the standard deviation 8.8 mm, the median 11.0 mm, and the range 6.0–40.8 mm. The median was close to the intended initial cut position of approximately 10 mm above the calyx, but the long upper tail shows that separation was not consistently achieved at this location. These measurements describe the initial field-detachment operation; they do not establish that every fruit was ready for packing without further trimming.

**Figure 10:**
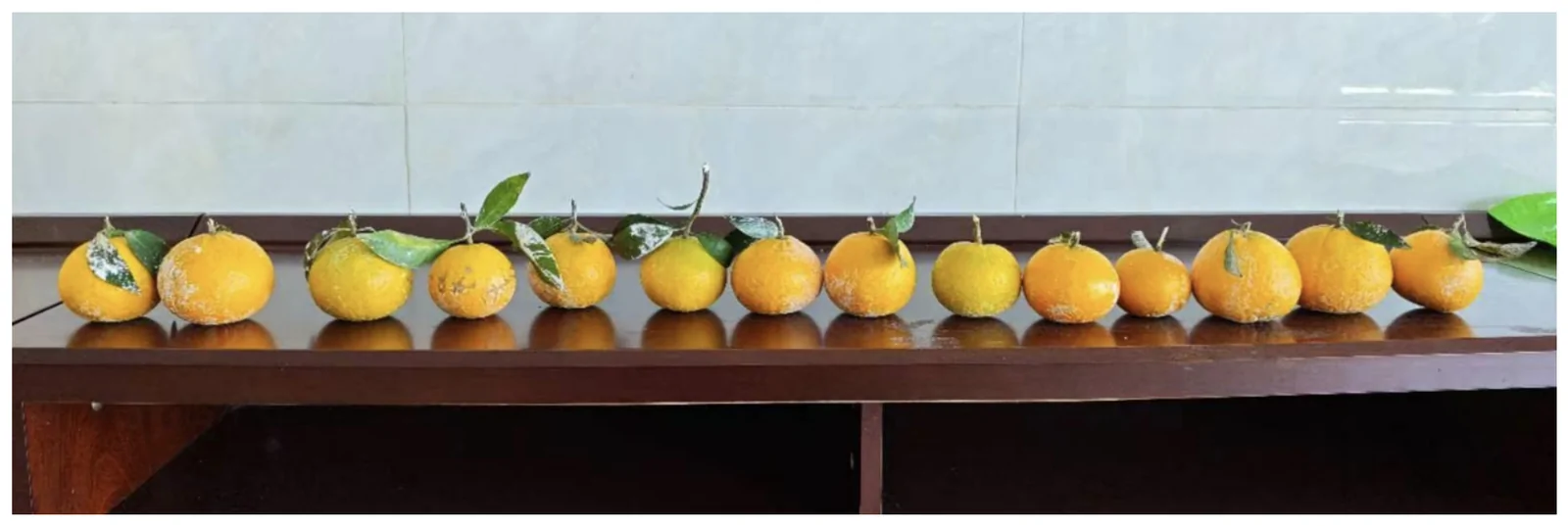
Harvested fruits illustrating variation in residual pedicel length after the initial detachment operation.

Figure 10 shows variation in retained pedicel length. Improving closure-plane placement and engagement stability should help the primary cutting edges meet the intended pedicel region more consistently.

## 6. Discussion

The main result is the field operation of a compact mechanism combining opposing C-ring engagement, primary cutting, optional wrist rotation, and passive fruit reception. Among the 85 recovered engaged outcomes, 73 met the visual acceptability criterion. The two damaging failure modes were equally frequent: six mis-cuts and six punctures. Mis-cuts were more severe because they transected the fruit body. These observations make closure-plane placement and ring-tip interaction the immediate design priorities.

The design builds on existing efforts to move separation forces towards the stalk or peduncle (Table 4). Kondo et al. [17] had already demonstrated a way to handle unknown peduncle orientations using main-stem guidance and sensing. Wang et al. [18] likewise addressed variable stalk orientation in citrus through a bite-mode mechanism and posture selection. The present contribution is therefore the particular integration of paired C-rings and staged pedicel separation with a receiving net for individual citrus fruit. Its approach does not require explicit pedicel-axis estimation, but the fruit and pedicel must still be positioned within the mechanism’s usable opening and cutting plane.

Within citrus harvesting, Xiao et al. [10] used flexible fingers to secure the fruit before rotating the end-effector to cut the stalk with a blade between the fingers. The C-ring design instead engages the pedicel without using a fruit-body clamp to stabilize cutting. This shifts the design emphasis from controlling grip contact to maintaining clearance between the closing rings and the peel. Parsa et al. [14] provide a complementary stem-handling approach: their strawberry tool clears obstructions with separator fingers and retains the cut stem for transfer to a punnet. The present mechanism uses a receiving net rather than retaining the cut stem; access to the closure region is arranged by the operator.

Reception also distinguishes integrated harvesting mechanisms. Zhang et al. [28] used vacuum attachment with rotational and, when needed, pulling motion for apple detachment, followed by release at a separate dropping point onto a slope and screw conveyor. Here, the net receives the fruit directly beneath the cutting region. These arrangements place different demands on the transfer path and surrounding clearance. Zhang et al. also identified physical obstruction by branches and foliage as a barrier to picking, reinforcing the importance of evaluating canopy access alongside the separation mechanism.

Reducing the need for a separately oriented narrow cutter may simplify alignment and offer an efficiency benefit. The current trials did not measure a complete picking cycle or include a concurrent comparison device, so this remains an operational prospect. Similarly, the observed fruit condition demonstrates feasibility under the stated inspection criteria, rather than a comparative reduction in damage. Studies that examine damage after handling and ripening, such as Goulart et al. [29], illustrate why immediate surface inspection and later quality evaluation should be distinguished.

The contact model explains one aspect of the concave surface under prescribed normal loading. Its homogeneous material, frictionless interface, and fixed fruit support do not reproduce the full harvesting interaction. In particular, mis-cuts involve a displaced closure plane, punctures involve local fracture, and grazes involve tangential motion. The simulation and field observations consequently serve complementary purposes: the former describes idealized normal response, while the latter identifies the damaging interactions that remain to be addressed.

Residual pedicel length was another limitation. The median of 11.0 mm was close to the positioning target, but a maximum of 40.8 mm indicates substantial variation in the separation location. Blade engagement, ring alignment, and the completion of primary cutting merit refinement before any claim of consistent cut quality. Storage studies are also needed to assess delayed peel damage and decay after harvesting [6,30].

Finally, these outcomes depend on operator selection of accessible fruit, manual alignment, and visual stage decisions. The detection score measures the supporting image-recognition module, while the field proportion describes recovered outcomes after engagement. Neither measures autonomous harvesting coverage across the entire canopy. Further work should combine improved ring-tip protection and closure-plane control with separation-state sensing, automated stage transitions, and canopy-aware approach planning. Larger trials and controlled comparisons would then establish how the integrated mechanism affects cycle time and fruit quality.

## 7. Conclusions

A bivalve-inspired C-ring end-effector was developed to engage and cut the pedicel of an individual citrus fruit without deliberately clamping the fruit body. Paired shell closure informed its structure and motion; fruit size and orchard access requirements determined the engineering dimensions. The device combines primary cutting with optional operator-controlled wrist rotation and passive reception in an elastic net.

Field evaluation under vision-assisted teleoperation produced 85 recovered engaged outcomes, of which 70 were intact and three had superficial grazes. The resulting acceptable-outcome proportion was 85.9% under the study’s visual criteria. Six mis-cuts and six punctures identified closure-plane placement and ring-tip contact as the main damage concerns. Residual pedicel lengths in 14 sampled fruits had a median of 11.0 mm and a range of 6.0–40.8 mm, indicating a need for more consistent cutting. The results support further development of the integrated mechanism, with emphasis on alignment, contact protection, primary-cut reliability, and assessment of performance across a broader range of orchard conditions.

## 8. Patents

Two Chinese utility model patents related to the work reported in this manuscript have been granted: “A Harvesting End-Effector” (CN224154723U, granted on 24 April 2026) and “A Harvesting Robot” (CN224139609U, granted on 21 April 2026). Beihang University is listed as the applicant for both patents.

## Figures and Tables

**Table 1:** Measured shell dimensions and estimated equatorial inner-profile radii. The ratio $R_{\mathrm{inner}}/\left(H/2\right)$ summarizes the profile proportions across specimens.

Specimen	*L* (mm)	*H* (mm)	*W* (mm)	*C* (mm)	*h* (mm)	$R_{\mathrm{inner}}$ (mm)	Ratio
1	61.5	42.2	20.4	40.9	10.7	24.9	1.18
2	72.9	52.2	23.6	47.0	11.9	29.2	1.12
3	175.9	120.4	59.3	115.7	28.9	72.4	1.20
Mean ± sample SD							1.17±0.04

**Table 2:** Structural and functional mapping from the biological reference to the end-effector. Numbers correspond to Figure 2; dashes denote features of an existing component rather than separately numbered parts.

No.	Biological Feature	Engineering Counterpart	Function
1	Two rigid valves	Two C-shaped rings	Opposing closure around the pedicel region
2	Hinge axis	Coaxial rotary joint	Rotation about a shared axis
3	Adductor muscles	Ring-driving servo motors	Actuation of opposing closure
–	Concave inner shell surface	Concave C-ring inner surface	Distribution of incidental normal contact
–	Valve meeting margins	Opposing C-ring cutting edges	Pedicel cutting during closure
4	No direct counterpart	Wrist-roll interface	Transmission of auxiliary wrist rotation
5	No direct counterpart	Elastic fruit-catching net	Reception and retention after detachment

**Table 3:** Recovered field outcomes after cutting and reception, stratified by fruit size. Type A: fruit-body mis-cut; Type B: ring-tip puncture; Type C: superficial graze.

Size	Assessed	Intact	Type A	Type B	Type C
Large	45	36	2	5	2
Small	40	34	4	1	1
Pooled	85	70	6	6	3

**Table 4:** Structural and functional comparison of related harvesting mechanisms.

Study	Target	Fruit Interaction	Stalk or Approach Requirement	Separation	Retention
Kondo et al. [17]	Tomato clusters	Peduncle held; fruit bodies not gripped	Main-stem access and guided search; prior peduncle orientation not required	Peduncle holding and cutting	Held peduncle, then release to tray
Feng et al. [12]	Cherry-tomato bunches	Stalk held	Locate bunch and align tool with stalk	Stalk holding and cutting	Stalk grasper and transport belt
Wang et al. [18]	Citrus fruit	Stalk-directed bite cutting	Posture selected for variable stalk orientation	Bite-mode stalk cutting	Not specified
Quaglia et al. [13]	Grape bunches	Peduncle held	Accessible peduncle within cutting and holding jaws	Underactuated holding and blade cutting	Peduncle retained by tool
Park et al. [15]	Tomatoes	Suction and guided contact	Pedicel enters cutting module; tool inclination affects access	Traction-assisted circular-saw cutting	Suction and transport modules
Parsa et al. [14]	Strawberry fruit	Stem held; target flesh not gripped	Stem aligned between jaws; separator fingers clear obstructions	Coupled stem gripping and cutting	Cut stem held for transfer
Xiao et al. [10]	Citrus fruit	Flexible fingers grip fruit	Fruit secured within fingers before tool rotation	Rotating blade cuts stalk	Fingers hold fruit for transfer
Zhao et al. [16]	Fruit-harvesting concepts	Enclosure or stem holding, depending on design	Scalable opening with alternative approach orientations	Direct cutting or stem-holding separation	Deployable enclosure
Present study	Individual citrus fruit	No deliberate fruit-body clamp; incidental contact possible	Pedicel crosses C-ring closing region; operator aligns closure plane	Opposing ring cut; wrist rotation if needed	Elastic net beneath rings

## Data Availability

The data supporting the findings of this study are available from the corresponding author on reasonable request, subject to commercial confidentiality restrictions.

## Abbreviations

The following abbreviations are used in this manuscript:

DOF	Degree of Freedom
FEA	Finite-Element Analysis
YOLO	You Only Look Once
CBAM	Convolutional Block Attention Module
